# From Laboratory to Real Clinical Practice: A Multidisciplinary Approach Towards the Next Probiotics

**DOI:** 10.3390/antibiotics15060595

**Published:** 2026-06-10

**Authors:** Matteo Pavoni, Giulia Fiorini, Ilaria Maria Saracino, Luigi Gatta, Raffaele Manta, John Holton, Natale Figura, Gabriella Massarenti, Chiara Leo, Beatrice Rosa, Cristina Marchesani, Stefano De Razza, Dino Vaira

**Affiliations:** 1 Department of Medical and Surgical Sciences, University of Bologna, Via Massarenti 9, 40138 Bologna, Italy; matteo.pavoni2@unibo.it (M.P.); saracinoilariamaria@gmail.com (I.M.S.); gabriella.massarenti@studio.unibo.it (G.M.); beatrice.rosa6@studio.unibo.it (B.R.); cristina.marchesani@studio.unibo.it (C.M.); stefano.derazza@gmail.com (S.D.R.); 2 Cardiovascular Medicine Unit, Heart, Chest and Vascular Department, IRCCS Azienda Ospedaliero-Universitaria di Bologna, University of Bologna, Via Massarenti 9, 40138 Bologna, Italy; giulia.fiorini@aosp.bo.it; 3 Management Staff Department, Area for Organizational Innovation, Complex Operational Unit for Outpatient and Diagnostic Demand Management, Tuscany North West Local Health Authority (ATNO), 56124 Pisa, Italy; gattalg@gmail.com; 4 Digestive Endoscopy Unit, Tuscany North West Local Health Authority (ATNO), “Spedali Riuniti” Hospital, 57124 Livorno, Italy; raffaelemanta4@gmail.com; 5 Department Natural Sciences (Microbiology), University of Middlesex, London NW4 4BT, UK; john.holton1@nhs.net; 6 Dartford and Gravesham NHS Trust, Darent Valley Hospital, Dartford DA2 8DA, Kent, UK; 7 Department of Biotechnology Chemistry and Pharmacy, University of Siena, 53100 Siena, Italy; natale.figura@unisi.it; 8 Polo d’Innovazione di Genomica, Genetica e Biologia SRL, Via Mazzieri, 05100 Terni, Italy; c.leo@pologgb.com

**Keywords:** *Helicobacter pylori*, sequencing, probiotics, eradication therapy

## Abstract

**Background and Aims**: *Helicobacter pylori* is a major cause of chronic gastritis and peptic ulcer disease. The increasing global spread of antibiotic-resistant strains, particularly against amoxicillin and clarithromycin, poses a significant challenge to eradication therapies. Moreover, treatment-related adverse effects, often linked to antibiotic-induced intestinal dysbiosis, frequently lead to a poor patient compliance; this, in turn, promotes the persistence of resistant bacterial populations. Probiotics may mitigate these effects and improve treatment adherence. This study aimed to assess the genomic safety of new probiotics intended for adjuvant use in *H. pylori* eradication regimens. **Methods**: Whole-genome sequencing was performed on three probiotic strains: one of *Lactobacillus acidophilus*, and two of *Bifidobacterium animalis* subsp. *lactis*. Genomes were compared with corresponding wild-type reference strains to identify genetic variations and detect mobile genetic elements. **Results**: Comparative genomic analysis revealed differences between selected and wild-type strains. Importantly, no plasmids or transposons were identified, suggesting a reduced theoretical risk of horizontal transfer of antimicrobial resistance determinants. Genomic findings were consistent with in vitro phenotypic observations. **Conclusions**: Whole-genome sequencing provided a robust assessment of the safety profile of these strains. The absence of transferable resistance elements supports their potential use as probiotic candidates to improve tolerability and adherence to *H. pylori* eradication therapies, contributing to more effective treatment outcomes.

## 1. Introduction

*Helicobacter pylori* is a Gram-negative, microaerophilic bacterium capable of colonizing the gastric mucosa, where it is implicated in the pathogenesis of chronic gastritis and peptic ulcer disease. Moreover, persistent infection represents a major etiological factor in the onset of gastric mucosa-associated lymphoid tissue (MALT) lymphoma and gastric adenocarcinoma [1]. The global prevalence of infection remains remarkably high, affecting more than half of the world’s population, with considerable geographic variability linked to socioeconomic and environmental factors [2]. Despite the widespread implementation of established eradication regimens, the management of *H. pylori* infection continues to pose significant clinical challenges. Conventional treatment strategies, which typically consist of proton pump inhibitors in combination with multiple antibiotics, have shown a gradual decline in efficacy over time. This reduction in therapeutic success is primarily driven by the global emergence and spread of antibiotic-resistant strains, particularly those exhibiting resistance to clarithromycin and metronidazole. As a result, there is a pressing need to explore and develop alternative or adjunctive therapeutic approaches to enhance eradication rates [3]. In this context, the occurrence of treatment-related adverse effects, frequently associated with antibiotic-induced intestinal dysbiosis, represents an additional clinically relevant issue. Antibiotic therapy can profoundly alter the composition and diversity of the gut microbiota, leading to gastrointestinal symptoms such as diarrhea, nausea, and abdominal discomfort. These adverse events often result in poor patient compliance and premature discontinuation of eradication regimens. Such interruptions may, in turn, facilitate the survival and clonal expansion of resistant bacterial populations, thereby contributing to a self-perpetuating cycle of therapeutic failure and increasing the overall burden of antimicrobial resistance [4].

Notably, *H. pylori* colonization is commonly associated with a reduction in microbial diversity, resulting in an altered microbial equilibrium consistent with dysbiosis. Furthermore, the co-administration of probiotics during antibiotic therapy may mitigate intestinal dysbiosis, thereby reducing gastrointestinal side effects and improving treatment adherence. In recent years, increasing attention has been directed toward the potential role of probiotics as an adjunct to conventional antibiotic therapy. Probiotics are live microorganisms that, when administered in adequate amounts, confer a health benefit on the host through multiple mechanisms, including competitive exclusion of pathogenic bacteria, production of antimicrobial compounds, enhancement of epithelial barrier function, and modulation of host immune responses. The co-administration of probiotics during antibiotic treatment has been shown to mitigate intestinal dysbiosis, reduce the incidence and severity of gastrointestinal side effects, and improve overall patient adherence to therapy.

Consistently, a growing body of evidence indicates that probiotic supplementation may enhance *H. pylori* eradication rates while simultaneously decreasing treatment-associated adverse effects, with greater efficacy observed when probiotics are administered both prior to and throughout the eradication regimen [5]. However, the effectiveness of probiotic interventions appears to be strain-specific, underscoring the importance of carefully selecting probiotic candidates based on their functional properties and clinical safety profiles.

In this context, the use of specific probiotic strains represents a particularly promising strategy, as it ensures their viability and functional activity even during concomitant antibiotic exposure. Such characteristics are essential for maintaining their beneficial effects throughout the treatment course. Accordingly, this study outlines a comprehensive and systematic framework for the selection of optimal probiotic strains, integrating key criteria such as functional efficacy, tolerance to antibiotic exposure, innovation, safety, and translational potential. Through this rigorous approach, we aim to identify probiotic candidates with the capacity to enhance therapeutic outcomes in *H. pylori* infection and to support the development of more effective and sustainable treatment strategies.

The primary objective of the present study was to perform a preclinical genomic safety characterization of selected probiotic strains intended for potential adjuvant use during *H. pylori* eradication therapy, in accordance with current EFSA recommendations for probiotic safety assessment [6].

## 2. Results

Following the selection of the most appropriate bacterial strains for the production of the probiotic, an NGS sequencing analysis was carried out.

A total number of 26,464,762 reads resulted from sequencing, with an average number of 5,292,952 reads per sample. Following the application of the software trimmomatic v0.36, a total of 24,749,594 reads passed the quality control, with an average number of reads per sample of 4,949,918. Table 1 shows the detailed number of reads and the mean Phred score before and after the trimming.

Overall, no contaminations were detected, with less than 5% of reads assigned to unexpected organisms (details are shown in Table 2).

The proportion of reads mapped to the reference genomes was 99%. These findings confirmed the high genomic consistency of the analysed strains and supported the reliability of downstream comparative analyses. The proportion of the reference genomes covered at least at 5× ranged between 99.59% and 99.99% with a median depth of 211–602×.

The Average Nucleotide Identity (ANI) was calculated between each sample and respective reference genome. The ANI analysis confirmed the taxonomic assignment of all strains while also supporting the presence of strain-level genomic differences relative to the parental reference genomes.

None of the samples was identified as a human pathogen using the program PathogenFinder v1.1. The probability of analyzed samples of being a human pathogen ranged between 0.20 and 0.23, and no pathogenic elements were found in the reconstructed consensus sequences. According to the PathogenFinder algorithm, probabilities closer to 1 are associated with a higher predicted pathogenic potential, whereas low probability scores support a non-pathogenic profile. As a reference, *Salmonella enterica* score corresponds to 0.94. In addition, none of the samples showed hit with at least 80% of identity and 70% of coverage with known virulence factors, included in the Virulence Factor Database (VFDB; Accessed on November 2023).

Therefore, the observed values were considered consistent with the safety profile expected for probiotic microorganisms intended for human use.

Through the obtained data, it was possible to point out that the wildtype genomes of the strains differ from those of the strains under study. Importantly, no plasmids, transposons, or other mobile genetic elements associated with horizontal gene transfer were identified. This finding is particularly relevant from a safety perspective, as it minimizes the theoretical risk of dissemination of antimicrobial resistance determinants to other microorganisms.

## 3. Discussion

*Helicobacter pylori* infection continues to represent a significant challenge in gastroenterological practice, with eradication failure remaining a frequent clinical concern. The success of current therapeutic regimens is primarily undermined by two closely interconnected factors: the increasing prevalence of antibiotic resistance and suboptimal patient adherence to treatment. In particular, the complexity and duration of eradication protocols, combined with the high incidence of gastrointestinal adverse effects (largely driven by antibiotic-induced disruption of the gut microbiota), often leads to poor compliance, thereby compromising therapeutic efficacy and further promoting the selection of resistant bacterial strains. In parallel, *H. pylori* infection itself profoundly reshapes the gastric microbiota, leading to reduced microbial diversity and pathogen dominance, while also exerting effects on the intestinal microbiome through alterations in the gastric environment and chronic inflammation [7]. Together, these processes support the existence of a bidirectional crosstalk between *H. pylori* and the gastrointestinal microbiota, resulting in a self-reinforcing cycle of dysbiosis that may favor bacterial persistence and reduce therapeutic efficacy, thus highlighting the usefulness of probiotic supplementation during eradication therapy.

The Maastricht VI Consensus provides indeed a strong recommendation (Level A2) for the use of specific probiotics to mitigate antibiotic-related side effects, thereby safeguarding patient compliance, while being more cautious regarding their role in directly increasing eradication rates (Level B2) [8]. This distinction highlights an ongoing debate regarding the primary mechanisms through which probiotics exert their beneficial effects in the context of *H. pylori* infection. Emerging evidence suggests that specific strains, such as *Lactobacillus* spp., *Bifidobacterium* spp., and *Saccharomyces boulardii*, not only have the ability to modulate microbiota composition counteracting or mitigating dysbiosis, but can also exert an inhibitory effect on *H. pylori* through competition for nutrients and adhesion sites, modulation of local and systemic immune responses, and the production of antimicrobial substances (bacteriocins) that inhibit pathogen growth and colonization [9,10].

Interestingly, a comprehensive meta-analysis of 45 randomized controlled trials demonstrated that while probiotic supplementation significantly improves eradication and reduces adverse events, it may not directly impact compliance rates in clinical trial settings. This suggests that the improved eradication success observed might also be attributed to the direct antagonistic effects of probiotics on *H. pylori*, rather than solely to better treatment adherence [11]. This apparent discrepancy raises the possibility that the observed therapeutic benefits may be driven, at least in part, by the direct antagonistic activity of probiotics against *H. pylori*, rather than solely by enhanced treatment adherence. Nevertheless, an important limitation of probiotic-based strategies lies in their susceptibility to concomitant antibiotic exposure, which may impair their viability and consequently reduce their functional efficacy during treatment. This aspect underscores the need for careful selection of probiotic strains capable of maintaining activity under antibiotic pressure.

In an in vitro study conducted by our group, five probiotic strains (*L. casei*, *L. paracasei*, *L. acidophilus*, *B. animalis* subsp. *lactis* and *S. thermophilus*) demonstrated direct antimicrobial activity against *Helicobacter pylori*, showing both bacteriostatic and bactericidal effects. The best results were obtained with *Lactobacillus* spp. [12]. Since Actinobacteria (*Bifidobacterium* spp.) tend to create transient colonies, which fluctuate in the gastric lumen and pass into the intestine, while Firmicutes (*Lactobacillus* spp. and *Streptococcus* spp.) seem capable to form stable colonies in the gastric mucosa [13], we hypothesized that *Lactobacillus* spp. and *S. thermophilus* can act directly in the stomach, while *B. lactis* may work in the intestine, preventing dysbiosis. Furthermore, as noted above, the bactericidal activity of probiotics against *H. pylori* is partially due to bacteriocins and, since their identity is not yet fully characterized, the use of a broad spectrum of bacteriocins rather than those produced by a single organism may provide a more effective adjunctive strategy in *H. pylori* treatment. Importantly, the present work should be considered a preclinical characterization study focused on the genomic safety and translational suitability of selected probiotic strains, rather than a clinical efficacy study. Based on these in vitro findings and the rationale described above, we chose *Lactobacillus acidophilus* and *Bifidobacterium animalis* subsp. *lactis*. Importantly, beyond the selection of two strains capable of tolerating antibiotic exposure, a key finding of our study was the absence of plasmids or transposable genetic elements, indicating a negligible risk of horizontal transfer of antibiotic resistance determinants.

With a focus on minimizing treatment failure, it is essential to develop co-adjuvants to therapy that provide clinicians with additional tools to prevent a drop in patient compliance due to the discomfort that may be caused by antibiotic therapy. Studying every component of eradication therapy in a systematic and standardized approach allows the implementation of probiotics in clinical practice to be made universally applicable. Although direct anti-*Helicobacter pylori* efficacy and patient outcomes were not evaluated in the present study, the obtained genomic data provide an essential preliminary framework for the future clinical development of these probiotic candidates. We also acknowledge that whole-genome sequencing alone cannot completely predict the biological behavior of probiotic strains in vivo, and that functional characterization remains essential for confirming clinical safety and efficacy.

## 4. Materials and Methods

Five putative probiotic strains, *Lactobacillus casei* DGDG, *Lactobacillus paracasei* LPC-S01, *Lactobacillus acidophilus* LA14, *Bifidobacterium animalis* subsp. *lactis* BL04, and *Streptococcus thermophilus* ST21, were initially chosen according to their ecological origin, their ability to withstand acidic and bile conditions, and their presumed capacity to persist within the gastric and/or intestinal environment [12].

The selected strains were subsequently subjected to an in vitro evaluation of growth kinetics, with particular emphasis on both proliferation rate and biomass production under increasing levels of antibiotic pressure. Specifically, bacterial cultures were exposed to progressively higher concentrations of amoxicillin and clarithromycin. Particular attention was devoted to tolerance against these two antibiotics, as they represent cornerstone agents in standard first-line eradication regimens for *H. pylori*. Strains exhibiting sustained growth and stable biomass yield despite antibiotic exposure were considered the most suitable candidates for further investigation. The purpose of this screening procedure was not to determine minimum inhibitory concentrations (MICs), but rather to identify probiotic candidates capable of maintaining stable growth performance under antibiotic exposure, a relevant characteristic for potential co-administration during eradication therapy.

Following the screening procedure, three strains—two belonging to the *Bifidobacterium animalis* subsp. *lactis* group and one to the *Lactobacillus acidophilus* group—displayed the most favorable profile in terms of growth performance and antibiotic resilience. These strains were subsequently deposited and registered at the Istituto Zooprofilattico Sperimentale della Lombardia e dell’Emilia-Romagna (IZSLER; www.izsler.it accessed on 10 February 2026) and assigned the new strain designations LAC6, BLA8, and BLC6.

**Figure 1:**
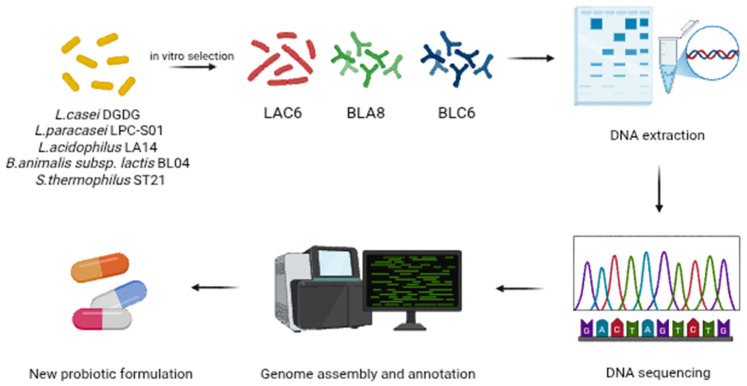
Study workflow.

In order to accurately characterize these bacterial strains, whole-genome sequencing methods were used to investigate their novelty compared to standard probiotic strains and their safety for patients. In Figure 1, the proposed workflow is represented.

### 4.1. DNA Extraction and Sequencing

Total genomic DNA was extracted from 500 µL of four resistant strains of *Bifidobacterium animalis* subsp. *lactis* and 500 µL of two resistant strains of *Lactobacillus* using the NucleoSpin Tissue Kit (Macherey-Nagel GmbH & Co. KG, Düren, Germany). The DNA extraction was performed following the manufacturer’s instructions. The DNA concentration was then assessed using the Qubit^®^ 4.0 Fluorometer and the DNA libraries were prepared in accordance with the Illumina Nextera XT DNA Library Prep Kit, following the manufacturer’s instructions (Illumina, San Diego, CA, USA). The libraries were then validated using the Fragment Analyzer™ High Sensitivity Small Fragment (Agilent Technologies, Santa Clara, CA, USA) and Qubit^®^ 4.0 Fluorometer. Finally, the libraries were loaded onto an Illumina MiSeq platform and sequenced as follows: BL04-A8-C6, LA14 A8, LA14 C6 with a 2 × 300 paired-end V3 chemistry, and samples BL04 A8, BL04 C6 and BLAC33 with a 2 × 150 paired-end V2 chemistry.

### 4.2. Genome Assembly and Annotation

The quality of the demultiplexed samples was checked using the program FastQC v0.11.9 [14] and the raw sequences were pre-processed based on quality using the tool Trimmomatic v0.36 [15]. The reads were then aligned to the respective reference sequences (Accession numbers: GCF_000022705.1 and GCF_000389675.2 for BL04 and LA14 samples, respectively) using the program bwa mem v0.7.17 [16]. The duplicated reads were marked using the package MarkDuplicates of the program Picard v2.18. The variants were called using the tool HaplotypeCaller of the software GATK v4.0.1 and the genotypes were jointly called using the package GenotypeGVCF of the software GATK v4.0.1 [17]. The variants were then filtered based on quality using the tool VariantFiltration and were annotated using the program snpEff v4_3 [18]. Particular attention was devoted to the identification of mobile genetic elements, transferable antimicrobial resistance determinants, virulence factors and pathogenicity-associated sequences, in line with EFSA recommendations for the safety assessment of microorganisms intentionally used in the food and probiotic chain.

## 5. Conclusions

The obtained data provides a comprehensive overview of the safety, novelty, and reliability of the probiotic strains intended to be used. In our opinion, as probiotics are increasingly being used as an alternative to pharmaceuticals, it is essential to have a scientific and consistent methodology for developing this type of product. The advantages of a multidisciplinary approach, from basic biology to microbiology and genetics, provide clinicians with preliminary scientific evidence supporting the safety and potential translational applicability of selected probiotic strains.

The selection of probiotic strains in the laboratory provides an initial screening of the various options by observing the activity of interest in vitro.

Sequencing provides an objective and clear picture of potential safety issues. It also allows confirmation of phenotypic observations obtained in the laboratory and a deeper understanding of the results.

## Figures and Tables

**Table 1:** Number of reads, mean Phred score and total base pairs per sample before and after trimming.

**Before Trimming**			
**Sample Name**	Number of reads	Mean Phred quality	Total base pairs
**BL04-A8**	3,858,014	26.26	577,037,833
**BL04-C6**	5,074,678	26.47	757,572,098
**LA14-C6**	6,207,142	27.00	1,351,860,475
**BL04-Wild type**	4,832,222	21.06	1,500,330,658
**LA14-Wild type**	6,492,706	27.29	1,530,334,493
**After Trimming**			
**Sample Name**	Number of reads	Mean Phred quality	Total base pairs
**BL04-A8**	3,159,946	31.4	444,138,805
**BL04-C6**	4,263,998	31.5	598,891,046
**LA14-C6**	6,137,598	32.7	1,201,442,272
**BL04-Wild type**	4,768,000	30.6	1,432,396,766
**LA14-Wild type**	6,420,052	33.2	1,470,441,416

**Table 2:** Kraken2 program results. The percentage of classified and unclassified sequences, percentage of reads assigned to the expected species and the target species are shown.

Sample Name	% Sequences Classified	% Sequences Unclassified	% On Target	On Target Species
**BL04-A8**	99.92	0.08	99.72	*Bifidobacterium animalis* subsp. *lactis* (BL04)
**BL04-C6**	99.99	0.01	99.79	*Bifidobacterium animalis* subsp. *lactis* (BL04)
**LA14-C6**	99.87	0.013	99.18	*Lactobacillus acidophilus* (LA14)
**BL04-Wild type**	99.92	0.08	99.53	*Bifidobacterium animalis* subsp. *lactis* (BL04)
**LA14-Wild type**	99.87	0.013	99.04	*Lactobacillus acidophilus* (LA14)

## Data Availability

The original contributions presented in this study are included in the article. Further inquiries can be directed to the corresponding author.
